# Cognitive Barriers to COVID-19 Vaccine Uptake Among Older Adults

**DOI:** 10.3389/fmed.2021.756275

**Published:** 2021-10-26

**Authors:** Jonathan L. Chia, Andree Hartanto

**Affiliations:** School of Social Sciences, Singapore Management University, Singapore, Singapore

**Keywords:** cognitive barriers, vaccine uptake, vaccine hesitancy, older adults, COVID-19

The COVID-19 pandemic has resulted in tremendous loss of life. As of late-July 2021, there have been more than 191 million confirmed cases and over 4.1 million deaths recorded (1). Although most nations have developed some competency in COVID-19 containment (2–4), there are new challenges. The continual spread of COVID-19 has resulted in new variants (5–7). These new variants are posited to have a significantly higher transmissibility (8–10), with higher fatality rates (11, 12).

With complete eradication of the COVID-19 virus seeming highly unlikely, the shift for healthcare experts is now to reduce this pandemic into a state of mild disease endemicity (13–17). Vaccines play an essential role in this transition (14, 16).

Vaccines provide protection in a few crucial ways. In its most effective form, it serves to prevent natural infection; if immunity response following the vaccine does not prevent natural infection, it may still attenuate virus pathology, reducing the infectiousness and/or severity of disease symptoms (18). Only a while ago the world was racing towards developing a vaccine for this devastating disease (19), currently, with vaccines in hand, countries struggle to have significant portions of their population vaccinated (20–23). In order to achieve herd immunity through vaccines of 95 percent efficacy, calculations suggest about 63–76% of the population would have to be vaccinated (24). This range increases to 84–90% when including a safety margin (24). This safety margin is perhaps needful given a reduced efficacy against new COVID-19 variants observed in emerging reports (25–27).

The COVID-19 virus has been especially dangerous for older adults. Studies have shown the virus causing worse outcomes and having a higher mortality rate among older adults (28, 29). This is perhaps unsurprising given the known susceptibility of older adults to other infectious diseases (30, 31). Against the backdrop of global ageing trends, this is particularly concerning. The population of individuals aged 65 and over is growing faster than any other age group (32). Globally, there are over 727 million persons aged 65 years and over (33). With a sizable older adult population in many countries, and due to COVID-19's implications on the elderly, countries have consequently prioritised vaccinating older adults (34–36).

The collective impact of having substantial older adult populations, the elderly being more vulnerable to COVID-19, and the need to have high percentages of these senior vaccinated, beckons us to consider the barriers to inoculation among the elderly. Emerging research has identified demographic characteristics of older adults hesitant in receiving the COVID-19 vaccine. These older adults generally received less years of formal education and had less social contact (37, 38), a pattern also observed among the general population (39, 40). However, beyond demographic characteristics and factors, research on the specific beliefs held by these older adults have been sparse. Cognitive factors such as misconceptions and appraisal play a pivotal role in shaping health behaviours (41), including vaccination uptake. Efforts to modify an individual's health behaviour must take these cognitive factors into account, addressing those that are deemed relevant to the individual (42). Older adults are a population with specific characteristics (43), and with it, specific beliefs and cognitive barriers. Ergo, by assessing the beliefs of these hesitant older adults regarding COVID-19 and its vaccine, health messages and targeted inventions can be introduced more effectively. In the paucity of existing literature, we highlight emerging research and draw from literature on other vaccine hesitancies among older adults, so as to make sense of potential cognitive barriers to COVID-19 vaccine uptake among seniors.

Referring to previous literature, we posit that cognitive barriers to vaccination among the elderly may be explained through three broad categories: (1) misconceptions of virus treatment, (2) misconceptions of vaccines, and (3) misappraisal of infection threat.

## Misconceptions of Virus Treatment

The medical care of older adults often involves the management of chronic illnesses. Typically, therapeutic interventions for chronic illnesses consist of prescribed medication. As health problems tend to accumulate with age, older adults often have multiple comorbidities and are required to consume a variety of medication. Polypharmacy—the concomitant use of multiple drugs by a single individual—is an issue in present models of healthcare (44–46). The ubiquitous use of prescribed drugs in older adults health management may hence frame these older adults' understanding of viruses like the COVID-19, thinking that they may be treated through medications readily prescribed by doctors (47). Consequently, older adults with this misconception may not view vaccination as necessary. This notion aligns with schema theory, which posits that when new information becomes available (i.e., COVID-19 vaccination), a person tries to fit this new information into the pattern which he/she had used in the past to interpret information regarding a similar situation (48). Similarly, as the healthcare regimen of older adults often involve the reduction and management of *existing* conditions, the preventive role vaccinations play may not be readily understood. Some older adults may see the COVID-19 vaccine as means to cure or manage disease symptoms, needed only by individuals who have contracted the virus (47, 49). Continuing on this notion of cognitive framing, regular prescription of a healthy lifestyle to combat chronic illness may also lead some to think that they may turn to these as alternatives to the vaccine (50, 51). They may form a misbelief that robust immunity from the COVID-19 virus may be achieved through exercising, eating healthy, taking vitamins, or avoiding sick people (49, 52).

## Misconceptions of Vaccines

Vaccination side effects such as fever, chills, fatigue and swelling of lymph nodes are typical. The introduction of a foreign substance into the body elicits an immune response, and while certain individuals may not exhibit these side effects, its presence in mild forms does not warrant immediate concern. Immune response following vaccination is important, generating the appropriate antibodies to fight off future infections. While unpleasant, some older adults may view potential COVID-19 vaccine symptoms disproportionately negative (53). Some may misconstrue these side effects as “sickness,” and mislabel the COVID-19 vaccine as disease-causing (49, 54). Even when these side-effectives are appreciated as normative biological responses, some older adults may feel that their general frailty and/or ailments exclude them from vaccine suitability (47). Given the coverage on COVID-19 vaccine side effects such as blood clotting disorders from the Oxford AstraZeneca vaccine (55) and myocarditis associations with the Pfizer-BioNTech and Mordena vaccines (56, 57), this may perpetuate the misnomer that only fit individuals are suited for COVID-19 vaccines. Some older adults may also have the misconception that the COVID-19 vaccine is primarily for people travelling overseas and those who frequent crowded settings (47, 49), a misconception possibly derived from extensive media coverage on restricted air travel and safe distancing measures. While greater contact with others and travel does increase the likelihood of infection, infections do occur domestically and within small groups.

## Appraisal of Infection Threat

Perceived disease susceptibility and severity of health threat form two important tenets of health behaviour engagement. According to the Protection Motivation Theory (58), these two components underpin overall threat appraisal. Working in tandem with perceived coping efficacy, they result in either adaptive (e.g., adopting health behaviour) or maladaptive responses (i.e., denial of health threat).

Perceived susceptibility reflects the appraisal of subjective risk to a negative situation (59, 60), such as an infection. Motivation to forgo pleasurable behaviours and engage in inconvenient protective health behaviour is in part driven by greater perceptions of infection susceptibility (61–65). Over the course of the pandemic, countries have developed and implemented various measures aimed at containing COVID-19 infection cases. In countries where the infection curve has been flattened or have lower incidences, perception of infection susceptibility may be reduced, and these populations may not feel the pressure to be vaccinated (21, 54). Previous research on the influenza vaccine have also found past episodes of influenza infection tended to substantiate notions of disease susceptibility (47). A lack of past COVID-19 infections and low incidence of community infections may lead some to trivialise the probability of COVID-19 infection, and hence may not be willing to be vaccinated.

Perceived health threat severity may be evaluated through group comparisons. When considering one's vulnerability to a particular disease, a stereotypical image of a high-risk group emerges (66, 67). Following which, a process of social comparison occurs, assessing similarities and differences between this stereotypical group and oneself (68). Inaccurate inferences impaired by self-enhancement biases may shape perceived vulnerability towards infection. For example, older adults who did not identify as being old or frail were less likely to abide by government guidelines to protect against overheating, trivialising potentially severe health implications; this was despite them being considered at risk objectively (69). Similarly, older adults who did not identify as sickly and frail were found were found to refuse vaccination on grounds that they had generally been healthy (49, 54), despite being considered at risk objectively (28, 29).

Consequently, behaviour engagement are held to be more likely when an individual perceives oneself to be faced with a health threat to which he/she is susceptible and which is perceived to be severe (41, 58).

## Conclusion and Implications

COVID-19 fatigue has certainly been experienced by many, yet, vigilance and tenacity is still needful. Given the sizable population of older adults and their vulnerability to the disease, having a large percentage of these older adults vaccinated is fundamental. Recent research suggest that the willingness to vaccinate against COVID-19 might be systematically underestimated, and introducing certain virus-related information (i.e., information about herd immunity) significant increases vaccination willingness (70). This highlights the modulating capability of nuanced messaging on vaccination willingness. Identifying subpopulations and demographic descriptors of unvaccinated seniors are important, however, it is equally important to understand the misconceptions, concerns and fears *specific* to this group of elderly individuals. When addressing the aforementioned barriers, it is also necessary to consider appropriate and accessible channels. Previous research has suggested that targeted messaging and intervention may be more effective when introduced through a family doctor (54), grassroots volunteers from the community using a multi-component approach [i.e., home visits, telephone and leaflets reminders; (71, 72)], and through traditional media such as television and newspapers (73). Further, as older adults may be more susceptible to misinformation (74, 75), establishing that messages through said channels are verified—whether through government (76) or journalist intervention (77)—and education on digital literacy (78, 79) may serve as potential counters.

## Author Contributions

The first draft of the manuscript was prepared by JC. Both authors contributed to manuscript revision, read, and approved the submitted version.

## Funding

This research was supported by a grant awarded to AH by Singapore Management University through research grants from the Ministry of Education Academy Research Fund Tier 1 (20-C242-SMU-001) and Lee Kong Chian Fund for Research Excellence.

## Conflict of Interest

The authors declare that the research was conducted in the absence of any commercial or financial relationships that could be construed as a potential conflict of interest.

## Publisher's Note

All claims expressed in this article are solely those of the authors and do not necessarily represent those of their affiliated organizations, or those of the publisher, the editors and the reviewers. Any product that may be evaluated in this article, or claim that may be made by its manufacturer, is not guaranteed or endorsed by the publisher.

## References

[1] World Health Organization. WHO Coronavirus Disease (COVID-19) Dashboard (2021, July 23). Available online at: https://covid19.who.int/ (accessed August 9, 2021).

[2] Arshed N Meo MS Farooq F. Empirical assessment of government policies and flattening of the COVID19 curve. J Public Affairs. (2020) 20:e2333. 10.1002/pa.2333 32904924 PMC7460975

[3] Lee D Heo K Seo Y Ahn H Jung K Lee S. Flattening the curve on COVID-19: South Korea's measures in tackling initial outbreak of coronavirus. Am J Epidemiol. (2021) 190:496–505. 10.1093/aje/kwaa217 33106843 PMC7665306

[4] Karatayev VA Anand M Bauch CT. Local lockdowns outperform global lockdown on the far side of the COVID-19 epidemic curve. Proc Nat Acad Sci. (2020) 117:24575–80. 10.1073/pnas.2014385117 32887803 PMC7533690

[5] Duong D. What's important to know about the new COVID-19 variants? Can Med Assoc J. (2021) 193:E141–2. 10.1503/cmaj.1095915 33667185 PMC7954556

[6] Mahase E. COVID-19: What new variants are emerging and how are they being investigated? BMJ. (2021) 372:n158. 10.1136/bmj.n158 33462092

[7] Wise J. COVID-19: The E484K mutation and the risks it poses. BMJ. (2021) 372:n359. 10.1136/bmj.n359 33547053

[8] Campbell F Archer B Laurenson-Schafer H Jinnai Y Konings F Batra N. Increased transmissibility and global spread of SARS-CoV-2 variants of concern as at June 2021. Eurosurveillance. (2021) 26:2100509. 10.2807/1560-7917.ES.2021.26.24.2100509 34142653 PMC8212592

[9] Coutinho RM Marquitti FMD Ferreira LS Borges ME da Silva RLP Canton O. Model-based evaluation of transmissibility and reinfection for the P. 1 variant of the SARS-CoV-2. MedRxiv [Preprint]. (2021). 10.1101/2021.03.03.21252706 PMC9053218 35602219

[10] Kumar V Singh J Hasnain SE & Sundar D. Possible link between higher transmissibility of Alpha, Kappa and Delta variants of SARS-CoV-2 and increased structural stability of its spike protein and hACE2 affinity. Int J Mol Sci. (2021) 22:9131. 10.3390/ijms22179131 34502041 PMC8431609

[11] Challen R Brooks-Pollock E Read JM Dyson L Tsaneva-Atanasova K Danon L. Risk of mortality in patients infected with SARS-CoV-2 variant of concern 202012/1: matched cohort study. BMJ. (2021) 372:n579. 10.1136/bmj.n579 33687922 PMC7941603

[12] Davies NG Jarvis CI Edmunds WJ Jewell NP Diaz-Ordaz K Keogh RH. Increased mortality in community-tested cases of SARS-CoV-2 lineage B.1.1.7. Nature. (2021) 593:270–4. 10.1038/s41586-021-03426-1 33723411 PMC9170116

[13] Hunter P. The spread of the COVID-19 coronavirus: health agencies worldwide prepare for the seemingly inevitability of the COVID-19 coronavirus becoming endemic. EMBO Rep. (2020) 21:e50334. 10.15252/embr.202050334 32181577 PMC7132190

[14] Lavine JS Bjornstad ON Antia R. Immunological characteristics govern the transition of COVID-19 to endemicity. Science. (2021) 371:741–5. 10.1126/science.abe6522 33436525 PMC7932103

[15] Tkachenko AV Maslov S Wang T Elbanna A Wong GN Goldenfeld N. Stochastic social behavior coupled to COVID-19 dynamics leads to waves, plateaus and an endemic state. medRxiv [Preprint]. (2021). 10.1101/2021.01.28.21250701 PMC8670744 34747698

[16] Torjesen I. Covid-19 will become endemic but with decreased potency over time, scientists believe. BMJ. (2021) 372:n494. 10.1136/bmj.n494 33602668

[17] Zaman M Tiong D Saw J Zaman S Daniels MJ. Sustainable resumption of cardiac catheterization laboratory procedures, and the importance of testing, during endemic COVID-19. Curr Treat Options Cardiovasc Med. (2021) 23:1–13. 10.1007/s11936-021-00901-w 33642850 PMC7897736

[18] Halloran ME Longini IM Struchiner CJ Longini IM. Design and Analysis of Vaccine Studies. Vol. 18. New York, NY: Springer (2010).

[19] Burgos RM Badowski ME Drwiega E Ghassemi S Griffith N Herald F. The race to a COVID-19 vaccine: opportunities and challenges in development and distribution. Drugs Context. (2021) 10:1–10. 10.7573/dic.2020-12-2 33643421 PMC7889064

[20] BBC News. What's Gone Wrong With Australia's Vaccine Rollout? (2021, June 17). Available online at: https://www.bbc.com/news/world-australia-56825920 (accessed August 9, 2021).

[21] Lee YN. Charts Show Asia is far Behind the U.S. and Europe in Covid Vaccinations. CNBC (2021, June 3). Available online at: https://www.cnbc.com/2021/06/04/covid-vaccine-hesitancy-in-asia-which-lags-us-europe-as-cases-surge.html (accessed August 9, 2021).

[22] Tan Y. COVID-19: What Went Wrong in Singapore and Taiwan? BBC News (2021, May 20). Available online at: https://www.bbc.com/news/world-asia-57153195 (accessed August 9, 2021).

[23] WHO Regional Office for Europe. Slow Vaccine Roll-Out Prolonging Pandemic (2021, March 31). Available online at: https://www.euro.who.int/en/media-centre/sections/press-releases/2021/slow-vaccine-roll-out-prolonging-pandemic (accessed August 9, 2021).

[24] Kadkhoda K. Herd Immunity to COVID-19: alluring and Elusive. Am J Clin Pathol. (2021) 155:471–2. 10.1093/ajcp/aqaa272 33399182 PMC7929447

[25] Abu-Raddad LJ Chemaitelly H Butt AA. Effectiveness of the BNT162b2 Covid-19 vaccine against the B. 1.1. 7 and B. 1.351 variants. N Engl J Med. (2021) 385:187–9 10.1056/NEJMc2104974 33951357 PMC8117967

[26] Madhi SA Baillie V Cutland CL Voysey M Koen AL Fairlie L. Efficacy of the ChAdOx1 nCoV-19 Covid-19 vaccine against the B. 1.351 variant. N Engl J Med. (2021) 384:1885–98. 10.1056/NEJMoa2102214 33725432 PMC7993410

[27] Lopez Bernal J Andrews N Gower C Gallagher E Simmons R Thelwall S. Effectiveness of Covid-19 vaccines against the B.1.617.2 (Delta) Variant. N Engl J Med. (2021) 385:585–94. 10.1056/nejmoa2108891 34289274 PMC8314739

[28] Nikolich-Zugich J Knox KS Rios CT Natt B Bhattacharya D Fain MJ. SARS-CoV-2 and COVID-19 in older adults: what we may expect regarding pathogenesis, immune responses, and outcomes. Geroscience. (2020) 42:505–14. 10.1007/s11357-020-00186-0 32274617 PMC7145538

[29] Shahid Z Kalayanamitra R McClafferty B Kepko D Ramgobin D Patel R. COVID-19 and older adults: what we know. J Am Geriatr Soc. (2020) 68:926–9. 10.1111/jgs.16472 32255507 PMC7262251

[30] Gardner ID. The effect of aging on susceptibility to infection. Rev Infect Dis. (1980) 2:801–10. 10.1093/clinids/2.5.801 6763306

[31] Schneider EL. Infectious diseases in the elderly. Ann Intern Med. (1983) 98:395–400. 10.7326/0003-4819-98-3-395 6830081

[32] United Nations. World Population Ageing 2019. (2020). Available online at: https://www.un.org/en/global-issues/ageing (accessed August 9, 2021).

[33] United Nations. World Population Ageing 2020 Highlights: Living Arrangements of Older Persons. (2021). Available online at: https://www.un.org/development/desa/pd/sites/www.un.org.development.desa.pd/files/undesa_pd-2020_world_population_ageing_highlights.pdf (accessed August 9, 2021).

[34] Centers for Disease Control and Prevention. How CDC is Making COVID-19 Vaccine Recommendations. (2021, May 14). Available online at: https://www.cdc.gov/coronavirus/2019-ncov/vaccines/recommendations-process.html (accessed August 10, 2021).

[35] Ko Y Lee J Kim Y Kwon D Jung E. COVID-19 vaccine priority strategy using a heterogenous transmission model based on maximum likelihood estimation in the Republic of Korea. Int J Environ Res Public Health. (2021) 18:6469. 10.3390/ijerph18126469 34203821 PMC8296292

[36] Ministry of Health. Covid-19 Vaccination Brought Forward for All Seniors; Extended to Essential Services Personnel and Higher Risk Groups. (2021, March 8). Available online at: https://www.moh.gov.sg/news-highlights/details/covid-19-vaccination-brought-forward-for-all-seniors-extended-to-essential-services-personnel-and-higher-risk-groups (accessed August 9, 2021).

[37] Malani PN Solway E Kullgren JT. Older adults' perspectives on a COVID-19 vaccine. JAMA Health Forum. (2020) 1:e201539. 10.1001/jamahealthforum.2020.1539 36218482

[38] Tan M Straughan PT Lim W Cheong G. Special Report on COVID-19 Vaccination Trends Among Older Adults in Singapore. Singapore Management University, Centre for Research on Successful Ageing (2021). Available online at: https://ink.library.smu.edu.sg/rosa_reports/5/ (accessed August 9, 2021).

[39] Fisher KA Bloomstone SJ Walder J Crawford S Fouayzi H Mazor KM. Attitudes toward a potential SARS-CoV-2 vaccine: a survey of US adults. Ann Intern Med. (2020) 173:964–73. 10.7326/M20-3569 32886525 PMC7505019

[40] Robertson E Reeve KS Niedzwiedz CL Moore J Blake M Green M. Predictors of COVID-19 vaccine hesitancy in the UK household longitudinal study. Brain Behav Immun. (2021) 94:41–50. 10.1016/j.bbi.2021.03.008 33713824 PMC7946541

[41] Conner M. Cognitive determinants of health behavior. In: Handbook of Behavioral Medicine. New York, NY: Springer. (2010). p. 19–30.

[42] Elder JP Ayala GX Harris S. Theories and intervention approaches to health-behavior change in primary care. Am J Prev Med. (1999) 17:275–84. 10.1016/S0749-3797(99)00094-X 10606196

[43] Wang J Zhang Y Long S Fu X Zhang X Zhao S. Non-EPI vaccine hesitancy among chinese adults: a cross-sectional study. Vaccines. (2021) 9:772. 10.3390/vaccines9070772 34358188 PMC8310190

[44] Fincke BG Snyder K Cantillon C Gaehde S Standring P Fiore L. Three complementary definitions of polypharmacy: methods, application and comparison of findings in a large prescription database. Pharmacoepidemiol Drug Saf. (2005) 14:121–8. 10.1002/pds.966 15386712

[45] Morin L Johnell K Laroche ML Fastbom J Wastesson JW. The epidemiology of polypharmacy in older adults: register-based prospective cohort study. Clin Epidemiol. (2018) 10:289. 10.2147/CLEP.S153458 29559811 PMC5856059

[46] Planton J Edlund BJ. Strategies for reducing polypharmacy in older adults. J Gerontol Nurs. (2010) 36:8–12. 10.3928/00989134-20091204-03 20047247

[47] Teo LM Smith HE Lwin MO Tang WE. Attitudes and perception of influenza vaccines among older people in Singapore: a qualitative study. Vaccine. (2019) 37:6665–72. 10.1016/j.vaccine.2019.09.037 31542261 PMC7130882

[48] Axelrod R. Schema theory: an information processing model of perception and cognition. Am Polit Sci Rev. (1973) 67:1248–66. 10.2307/1956546

[49] Cummings CL Kong WY Orminski J. A typology of beliefs and misperceptions about the influenza disease and vaccine among older adults in Singapore. PLoS ONE. (2020) 15:e0232472. 10.1371/journal.pone.0232472 32374754 PMC7202625

[50] Chen CY Gan P How CH. Approach to frailty in the elderly in primary care and the community. Singapore Med J. (2018) 59:240. 10.11622/smedj.2018052 29799055 PMC5966632

[51] Zaleski AL Taylor BA Panza GA Wu Y Pescatello LS Thompson PD. Coming of age: considerations in the prescription of exercise for older adults. Methodist Debakey Cardiovasc J. (2016) 12:98. 10.14797/mdcj-12-2-98 27486492 PMC4969034

[52] Telford R Rogers A. What influences elderly peoples' decisions about whether to accept the influenza vaccination? A qualitative study. Health Educ Res. (2003) 18:743–53. 10.1093/her/cyf059 14654506

[53] Cornford CS Morgan M. Elderly people's beliefs about influenza vaccination. Br J Gen Pract. (1999) 49:281–4. 10736905 PMC1313393

[54] Fadda M Suggs LS Albanese E. Willingness to vaccinate against Covid-19: a qualitative study involving older adults from Southern Switzerland. Vaccine X. (2021) 8:100108. 10.1016/j.jvacx.2021.100108 34308329 PMC8279929

[55] Mahase E. Covid-19: WHO says rollout of AstraZeneca vaccine should continue, as Europe divides over safety. BMJ. (2021) 372:n728. 10.1136/bmj.n728 33727218

[56] Rosner CM Genovese L Tehrani BN Atkins M Bakhshi H Chaudhri S. Myocarditis temporally associated with COVID-19 vaccination. Circulation. (2021) 144:502–5. 10.1161/CIRCULATIONAHA.121.055891 34133885 PMC8340723

[57] Starekova J Bluemke DA Bradham WS Grist TM Schiebler ML Reeder SB. Myocarditis associated with mRNA COVID-19 vaccination. Radiology. (2021) 211430. 10.1148/radiol.2021211430. [Epub ahead of print]. 34282971 PMC8574056

[58] Rogers RW. A protection motivation theory of fear appeals and attitude change1. J Psychol. (1975) 91:93–114. 10.1080/00223980.1975.9915803 28136248

[59] Becker MH. The health belief model and sick role behavior. Health Educ Monogr. (1974) 2:409–19. 10.1177/1090198174002004072214655

[60] Venema T Pfattheicher S. Perceived susceptibility to COVID-19 infection and narcissistic traits. Personal Individ Differ. (2021) 175:110696. 10.1016/j.paid.2021.110696 33558779 PMC7857009

[61] Aiken LS Gerend MA Jackson KM Ranby KW. Subjective risk and health-protective behavior: prevention and early detection. In: Baum A Revenson TA Singer J, editors. Handbook of Health Psychology. New York, NY: Psychology Press. (2012). p. 113–45.

[62] Brewer NT Chapman GB Gibbons FX Gerrard M McCaul KD Weinstein ND. Meta-analysis of the relationship between risk perception and health behavior: the example of vaccination. Health Psychol. (2007) 26:136. 10.1037/0278-6133.26.2.136 17385964

[63] Ferrer RA Klein WM. Risk perceptions and health behavior. Curr Opin Psychol. (2015) 5:85–9. 10.1016/j.copsyc.2015.03.012 26258160 PMC4525709

[64] Leung GM Quah S Ho LM Ho SY Hedley AJ Lee HP. A tale of two cities: community psychobehavioral surveillance and related impact on outbreak control in Hong Kong and Singapore during the severe acute respiratory syndrome epidemic. Infect Control Hosp Epidemiol. (2004) 25:1033–41. 10.1086/502340 15636289

[65] Lau JT Kim JH Tsui HY Griffiths S. Anticipated and current preventive behaviors in response to an anticipated human-to-human H5N1 epidemic in the Hong Kong Chinese general population. BMC Infect Dis. (2007) 7:18. 10.1186/1471-2334-7-18 17359545 PMC1845150

[66] Weinstein ND. Unrealistic optimism about future life events. J Pers Soc Psychol. (1980) 39:806. 10.1037/0022-3514.39.5.80621058872

[67] Weinstein ND Klein WM. Resistance of personal risk perceptions to debiasing interventions. Health Psychol. (1995) 14:132. 10.1037/0278-6133.14.2.132 7789348

[68] Wood JV. What is social comparison and how should we study it? Pers Soc Psychol Bull. (1996) 22:520–37. 10.1177/0146167296225009

[69] Abrahamson V Wolf J Lorenzoni I Fenn B Kovats S Wilkinson P. Perceptions of heatwave risks to health: interview-based study of older people in London and Norwich, UK. J Public Health. (2009) 31:119–26. 10.1093/pubmed/fdn102 19052099

[70] Rieger MO. Willingness to vaccinate against COVID-19 might be systematically underestimated. Asian J Soc Health Behav. (2021) 4:81. 10.4103/shb.shb_7_21

[71] Ompad DC Galea S Vlahov D. Distribution of influenza vaccine to high-risk groups. Epidemiol Rev. (2006) 28:54–70. 10.1093/epirev/mxj004 16707648

[72] Thomas RE Lorenzetti DL. Interventions to increase influenza vaccination rates of those 60 years and older in the community. Cochrane Database Syst Rev. (2018) 5:CD005188. 10.1002/14651858.CD005188.pub4 29845606 PMC6494593

[73] Ahorsu DK Lin CY Pakpour AH. The association between health status and insomnia, mental health, and preventive behaviors: the mediating role of fear of COVID-19. Gerontol Geriatr Med. (2020) 6:2333721420966081. 10.1177/2333721420966081 33195740 PMC7594224

[74] Brashier NM Schacter DL. Aging in an era of fake news. Curr Dir Psychol Sci. (2020) 29:316–23. 10.1177/0963721420915872 32968336 PMC7505057

[75] Vijaykumar S. Covid-19: Older adults and the risks of misinformation. The BMJ Opinion (2020). 19p. Available online at: https://blogsbmicom/bmi/2020/03/13/covid-19-older-adults-and-the-risks-of-misinformation/Accessed.

[76] Lee H Lee T. From contempt of court to fake news: public legitimisation and governance in mediated Singapore. Media Int Aust. (2019) 173:81–92. 10.1177/1329878X19853074

[77] Haque MM Yousuf M Alam AS Saha P Ahmed SI Hassan N. Combating Misinformation in Bangladesh: roles and responsibilities as perceived by journalists, fact-checkers, and users. Proc ACM Hum Comput Int. (2020) 4:1–32. 10.1145/3415201

[78] Moore RC Hancock JT. Older adults, social technologies, and the coronavirus pandemic: challenges, strengths, and strategies for support. Soc Media Soc. (2020) 6:2056305120948162. 10.1177/2056305120948162

[79] Seo H Blomberg M Altschwager D Vu HT. Vulnerable populations and misinformation: a mixed-methods approach to underserved older adults' online information assessment. New Media Soc. (2021) 23:2012–33. 10.1177/1461444820925041

